# Advanced Glycation End Product Accumulation in Subjects with Open-Angle Glaucoma with and without Exfoliation

**DOI:** 10.3390/antiox9080755

**Published:** 2020-08-15

**Authors:** Tomoki Shirakami, Mikihiro Yamanaka, Jo Fujihara, Yotaro Matsuoka, Yuko Gohto, Akira Obana, Masaki Tanito

**Affiliations:** 1 Department of Ophthalmology, Shimane University Faculty of Medicine, Izumo 693-8501, Japan; t-shira@med.shimane-u.ac.jp; 2 Laboratory of Food and Regulation Biology, School of Agriculture, Tokai University, Kumamoto 862-8652, Japan; yamanaka.mikihiro@tsc.u-tokai.ac.jp; 3 Division of Ophthalmology, Matsue Red Cross Hospital, Matsue 690-8506, Japan; aoiroelephant@hotmail.co.jp (J.F.); ymatsu@med.shimane-u.ac.jp (Y.M.); 4 Department of Ophthalmology, Seirei Hamamatsu General Hospital, Hamamatsu 430-8558, Japan; yukogo@sis.seirei.or.jp (Y.G.); obana@sis.seirei.or.jp (A.O.)

**Keywords:** advanced glycation end products (AGEs), skin autofluorescence (sAF), AGEs Sensor, primary open-angle glaucoma, pseudoexfoliation syndrome, exfoliation glaucoma, oxidative stress

## Abstract

Advanced glycation end products (AGEs), which are the products of a non-enzymatic reaction between reducing sugars and other macromolecules, are critical in aging, as well as metabolic and degenerative diseases. To assess the involvement of AGEs in glaucoma, skin autofluorescence (sAF) level, which is a measurement of AGEs’ accumulation, was compared among Japanese patients with glaucoma (316 with primary open-angle glaucoma (PG) and 127 exfoliation syndrome and glaucoma (EG)) and controls (133 nonglaucomatous controls) (mean age 71.6 ± 12.8 years, 254 men and 322 women). The sAF values were estimated from the middle fingertip using a 365 nm light-emitting diode for excitation and detection at 440 nm emission light. The estimated AGE values (arbitrary unit) were 0.56 ± 0.15, 0.56 ± 0.11, and 0.61 ± 0.11 in the control, PG, and EG groups, respectively (*p* < 0.0001, analysis of variance); and were significantly higher in the EG group than the control (*p* = 0.0007) and PG (*p* < 0.0001) groups. After adjustment for various demographic parameters by multivariate analyses, male sex (standard β = 0.23), EG (0.19), and diabetes (0.09) were associated with higher AGE levels; PG (−0.18) and smoking (−0.19) were associated with lower AGE levels. Age, visual acuity, intraocular pressure, glaucoma medications, lens status, and systemic hypertension were not associated with AGEs. The high AGE level in EG suggested that specific oxidation and glycation mechanisms underlie the glaucoma pathogenesis associated with pseudoexfoliation syndrome.

## 1. Introduction

Progressive optic neuropathy and visual field loss characterize glaucoma, a leading cause of irreversible blindness worldwide [1] including Japan [2]. Intraocular pressure (IOP) elevation is the primary risk factor of retinal ganglion cell (RGC) axon loss and apoptotic RGC death, and subsequent glaucomatous optic neuropathy [3]. In open-angle glaucoma (OAG) such as primary OAG (PG) and glaucoma secondary to pseudoexfoliation syndrome (EX), reduction of aqueous humor outflow at the trabecular meshwork (TM) is the main reason for the IOP elevation [4]. This can be the result of TM cells dysfunction and changes in the amount and quality of the extracellular matrix in the TM [5]. Previous clinical and experimental studies have reported that oxidative stress and inflammation were involved in the dysfunction of TM and the increase of aqueous outflow resistance [6,7,8].

We previously identified a significantly lower antioxidant capacity level and higher hydroxylinoleates, oxidation products of the linoleates, in blood samples from subjects with PG and EX as compared with those in control samples [9,10,11]. Interestingly, the lower level of blood antioxidant capacity was associated with higher IOP values in patients with glaucoma and control subjects [12] and with worse visual field defects in OAG [13,14]. More recently, we reported that supplementation with French maritime pine bark/bilberry fruit extracts, which are natural antioxidants compounds, could further reduce the IOP in Japanese patients with controlled PG [15].

Advanced glycation end products (AGEs) are various lipids, proteins, or nucleic acids that undergo glycation as a result of chronic exposure to glucose or other reducing sugars. AGEs accumulate in long-lived proteins such as collagen and have been indicated as components of oxidative stress generation in tissues [16,17,18]. From the late 1990s, AGEs have been measured in the human body noninvasively using the AGE Reader (DiagnOptics Technologies B.V., Groningen, The Netherlands) [19]. This device emits light-emitting diode light ranging from 300 to 420 nm on the forearm skin and detects the skin autofluorescence (sAF) ranging from 420 to 600 nm [20,21]. The results of enzyme-linked immunosorbent-based assays in patients with diabetes showed that specific AGEs had already accumulated in the skin before the onset of microangiopathy [22].

AGE formation and accumulation are associated with metabolic diseases such as diabetes mellitus [23,24,25] and physiologic changes such as aging [16,17,18,26,27]. Previous studies have reported that AGEs could also be involved in the incidence or progression of age-related chronic diseases, such as cardiovascular disease, heart failure, chronic kidney disease, and dementia and other neurodegenerative diseases [23,28,29,30]. AGEs have been presumed to be associated with the pathophysiologic processes of ocular diseases, such as cataract, diabetic retinopathy, and age-related macular degeneration [31,32,33]. In addition, glaucoma is an age-related chronic neurodegenerative disease affected by oxidative stress [34,35], and AGEs accumulate in the optic nerve head and RGC layer [36,37]. Therefore, we considered that AGEs are associated with the pathogenesis of glaucoma and measured the sAF levels to assess the possible involvement of AGEs in glaucoma.

## 2. Materials and Methods

### 2.1. Subjects

The current study adhered to the tenets of the Declaration of Helsinki. The institutional review board of the Matsue Red Cross Hospital approved the research conducted at the Matsue Red Cross Hospital (no. 303, issued on 21 September 2016) and at the Iinan Hospital (no. 309, issued on 18 November 2016). All participants provided written informed consent for inclusion in the study. Subjects were recruited consecutively at the Division of Ophthalmology, Matsue Red Cross Hospital, and the outpatient clinic at the Iinan Hospital. A total of 576 eyes of 576 Japanese subjects (254 men and 322 women, mean age ± standard deviation (SD) 71.6 ± 12.8 years) comprised the study population, i.e., subjects with PG (*n* = 316), exfoliation glaucoma (EG) (*n* = 127), and non-glaucomatous controls (*n* = 133). The participants underwent ophthalmologic examinations including measurement of the best-corrected visual acuity (BCVA) and IOP by Goldmann applanation tonometry, slit-lamp biomicroscopy, and funduscopy. One author (M.T.) measured the IOPs on the day of sample collection. In addition, the highest IOPs recorded in the previous medical records were investigated. The lens status (phakia/pseudophakia) and the number of glaucoma medications used by the patients were obtained from medical records. A fixed combination eyedrop was counted as two medications. The subjects were interviewed about current smoking habits and history of diabetes, and systemic hypertension. PG was diagnosed based on open iridocorneal angles bilaterally, the characteristic appearance of glaucomatous optic neuropathy such as enlargement of the optic disc cup or focal thinning of the neuroretinal rim, corresponding visual field defects in at least one eye, and no evidence of secondary glaucoma bilaterally. EG was diagnosed based on an open iridocorneal angle and characteristic pseudoexfoliation material deposits on the anterior capsule or pupillary margin in at least one eye. If both eyes were eligible, the eye with the worse mean deviation was included in the PG or EG group. Visual field defects were identified using the Humphrey Visual Field Analyzer (Carl Zeiss Meditec, Dublin, CA, USA). Control subjects were consecutively recruited from the patients who visited the ophthalmology outpatient clinics at the Matsue Red Cross Hospital, Matsue, Japan and the Iinan Hospital, Iinan, Japan. The controls, aged 20 years and older, had no clinical signs of glaucoma and did not use glaucoma medications, and the highest IOP of the control subjects was lower than 20 mmHg. The lens status was not considered. The eye of a study participant with the better BCVA was included in the analyses. If both eyes had the same BCVA, the right eye was included. For the PG, EG, and control groups, eyes with ocular diseases other than glaucoma and cataract and decimal BCVA worse than 0.01 were excluded. Since the association between diabetes and AGEs has been well documented [38,39,40], patients with severe diabetes that required insulin and was associated with the presence of diabetic retinopathy were excluded.

### 2.2. Measurement of AGEs in the Fingertip Skin

To estimate AGEs, the participants underwent measurements of the sAF levels using the AGEs Sensor (Air Water Biodesign Inc., Kobe, Japan). The sAF values obtained with the excitation and emission wavelengths of 365 nm and 440 nm, respectively, were used to estimate the levels of AGE accumulation. The obtained sAF levels are correlated positively with the level of the hyperglycemia-associated AGE, Nδ-(5-hydro-5-methyl-4-imidazolone-2-yl)-ornithine (MG-H1) [41]. The sAF is correlated with collagen-linked fluorescence (CLF) (excitation at 370 nm, emission at 440 nm) that degrades in skin biopsy specimen [19,42,43]. The levels of skin CLF reflect the actual levels of fluorescent and non-fluorescent AGEs such as pentosidine and MG-H1, and these levels of AGEs are correlated with well-known abundant AGEs such as Nε-(carboxymethyl)-lysine (CML) and Nε-(carboxyethyl)lysine [19,42,43]; therefore, the sAF is a good proxy for AGEs accumulation in tissues.

The finger clip feature of the device enables measurement of the intensity of the fluorescence from the middle finger of the non-dominant hand where the least skin melanin is present. Although the intensity of autofluorescence from veins is approximately 1.5-fold higher than that of skin, the influence of the veins is negligible in the measurement of the sAF using the AGEs Sensor, since the fingertips have only capillaries and no veins. Therefore, the fingertip is suitable to avoid non-specific skin fluorescence [41]. Trained examiners performed all measurements.

The measured AGEs were expressed in arbitrary units. According to our pilot study, the coefficient of variation and intraclass correlation coefficient (Cronbach’s α) of three repeated AGE measurements were 6.7 ± 7.3% and 0.938, respectively.

### 2.3. Statistical Analysis

The data are expressed as the means ± SD for continuous variables and in numbers and percentage for categorical variables. For the statistical analyses, the decimal BCVA was converted into the logarithm of the minimum angle of resolution. For comparisons among the three study groups, the differences in continuous data, age, BCVA, IOP, highest IOP, and number of glaucoma medications were calculated using one-way analysis of variance followed by post-hoc unpaired *t*-tests, and the differences in categorical data, i.e., sex, lens status, smoking, diabetes, and hypertension were calculated using the G-test followed by the post-hoc Fisher’s exact probability test. To correct for multigroup comparisons, *p* values of 0.0167 and 0.0033 for the unpaired *t* tests or Fisher’s exact probability test were considered to be significant at 5% and 1%, respectively, based on Bonferroni’s method. Possible correlations among AGEs and other parameters were assessed by linear regression analyses with Pearson’s correlation coefficient for continuous data and the unpaired t-test for categorical data. To adjust for differences in parameters among groups, possible associations among AGEs and various parameters were tested further using multiple regression analyses. All statistical analyses were calculated using JMP Pro statistical software version 14.2 (SAS Institute, Inc., Cary, NC, USA).

## 3. Results

The subject demographic data are shown in Table 1. In this dataset, all parameters differed significantly among three groups, except for the current smoking habits and IOP on the day of the AGE measurement. Table 2 and Figure S1 show the comparisons of AGEs among the three groups. The AGE levels of the EG group (0.61 ± 0.11) were significantly higher than those of the control group (0.56 ± 0.15, *p* = 0.0007) and those of the PG (0.56 ± 0.11, *p* < 0.0001) group, whereas the levels were equivalent in the control and PG groups (*p* = 0.5120).

Age was correlated positively with the AGEs (*r* = 0.11, *p* = 0.0063), while the BCVA, IOP on the day of the AGE measurement, highest IOP recorded, and number of glaucoma medications used were not correlated with the AGEs (Table 3). Male sex; pseudophakia; the presence of pseudoexfoliation, diabetes, and systemic hypertension; and no current smoking were associated with higher AGE levels than their corresponding groups (Table 4).

The correlations between AGEs and various parameters were analyzed by multiple regression analysis (Table 5) to adjust for possible confounding effects among parameters. Male sex (standard β = 0.23), EG (0.19), and diabetes (0.09) were correlated with higher AGE levels, while PG (−0.18) and smoking habits (−0.19) were correlated with lower AGE levels. Age, BCVA, IOP, highest IOP, glaucoma medications, lens status, and systemic hypertension were not associated with AGEs.

## 4. Discussion

We measured the AGE levels in patients with PG and EG and compared them with controls to assess the possible involvement of AGEs in the glaucoma pathogenesis and found that patients with EG had higher fingertip skin-measured AGE levels as compared with patents with PG and controls.

The AGEs Reader noninvasively measured AGEs in the human body by measuring the sAF levels on the forearm. When measuring the forearm skin, an approximate area of 4 cm^2^ of skin surface on the forearm was required to guard against the surrounding light, and the recommendation was that the measurement be performed in a dark place [21]. Any scars, visible vessels, abnormal lichenification, skin pigmentation, and use of skin creams can interfere with the measurement [44,45]. The reliability of the sAF measurements varied with a wide range of native skin colors within the Fitzpatrick skin phototypes from I (very pale skin, always burns) to IV (light brown skin, burns minimally) [46,47,48]; the forearm-based device could not evaluate participants with Fitzpatrick skin phototypes V (dark brown skin, very rarely burns) and VI (darkest brown skin, never burns) because darker skin pigmentation weakened the ultraviolet reflectance below 10%. The fingertip skin-based measurement was developed to compensate for these shortages. The fingertip contains less melanin that blocks light transmission and fewer veins that cause measurement errors [41]. The middle finger of the non-dominant hand has the lowest standard deviation of fluorescence intensity among the fingers, probably because this finger has the thinnest skin with the least frequency of use [41]. The clip on the device enables clamping of the fingertip that facilitated even pressure and was suitable for stable measurement of the fluorescence intensity [41]. Furthermore, because Japanese subjects have more melanin in their forearms than Caucasians, we adopted this AGEs sensor in our study [49,50].

Hondur et al. reported that the AGE levels were significantly higher in the aqueous humor and blood samples from patients with glaucoma than in samples from subjects without glaucoma [35]. Experimental studies have reported that AGEs and the receptor of AGEs accumulated in the glaucoma-related tissues such as the optic nerve head, RGC layer, and along with blood vessels. These accumulations contributed to glaucomatous optic neuropathy [36,37]. Several studies have assessed AGEs in patients with glaucoma [21,51,52]. Himori et al. reported that higher sAF levels were associated with worse visual fields in young patients with OAG (PG and normal tension glaucoma (NTG)) [51]. The same investigators reported an association between higher sAF levels and decreased optic nerve head blood flow in NTG [52]. Schweitzer et al. found that subjects with higher sAF levels had a higher prevalence of OAG (not limited to PG) than subjects with normal AGE levels [21]. In the current study, the AGEs were not elevated significantly in the PG group. The reasons for this discrepancy were unclear, but we speculated that the difference in demographics between the PG and controls groups (Table 1) affected the results. The glaucoma severity and race could be other possible reasons. Further study is needed to elucidate this discrepancy.

Our results clearly indicated that subjects with EG had higher AGE levels than those with PG and controls. The specific AGEs such as CML and pentosidine formed not only as a result of glycation, that is, the Maillard reaction, but also as a result of oxidation [53]. CML was detected in human lens capsules with EX [54]. Although patients with both PG and EG had reduced systemic antioxidant capacity as compared with controls [4,9,12,55], our previous studies have indicated that the oxidative pathways of PG and EG differ; comprehensive measurement of the serum levels of hydroxylinoleate isomers [56,57] have found that the enzymatic and singlet oxygen-mediated fatty acid oxidation could be the major oxidation pathways in patients with PG [10], whereas free radical-mediated oxidation could be a major oxidation pathway in EG [11]. During the Amadori rearrangement, reactive, intermediate products known as α-dicarbonyls or oxoaldehydes including 3-deoxyglucosone and methylglyoxal (MGO) were formed [41]. MGO has also been formed from non-oxidative mechanisms in anaerobic glycolysis [58] and from oxidative decomposition of polyunsaturated fatty acids [59]. Evidence for this came from in vitro experiments in which antioxidants resulted in a reduction in CML formation; metal-catalyzed oxidation of polyunsaturated fatty acids in the presence of proteins led to CML formation [60]. This result suggested that non-enzymatic lipid oxidation had a role in the formation of AGEs [61,62]. Accordingly, the difference in the underlying oxidation mechanisms between PG and EG could be associated with the significant elevation of AGEs in EG.

We found a significant association among high AGE levels and male gender, diabetes, and no smoking. Diabetes is a well-known factor that accelerates AGE levels [38,39,40]. There is no consensus about the gender difference in the AGE levels, which should be studied further. Previous reports have shown that tobacco smoking was associated with AGE formation [63,64], which differed from the current result. We counted only current smokers regardless of the history of smoking, and the number of smokers was small in all of the study groups. The present result regarding smoking could have some sample selection and sample size biases.

The current study had some limitations. Differences in the subject demographics such as age and sex among groups could have affected the results, although we tried to minimize the effect by multivariate analyses. Interview-based diagnosis of diabetes (i.e., absence of blood sugar data) and systemic hypertension (i.e., absence of actual blood pressure level), and no previous history of smoking could have weakened the detection power for involvement of those factors. The absence of parameters of glaucoma severity, such as visual field sensitivity and retinal nerve fiber thickness in the statistical analyses, was also was a study limitation.

## 5. Conclusions

In conclusion, the levels of AGEs in subjects with EG were higher than those with PG and non-glaucomatous control subjects. This result suggested that specific oxidation and glycation mechanisms underlie the pathogenesis of glaucoma associated with EX.

## Figures and Tables

**Table 1:** Demographic subject data.

Group	Control	PG	EG	*p* Value
*n*	133	316	127	
Age (years)				
Mean ± SD	70.1 ± 16.1	69.6 ± 11.8	78.2 ± 8.9	<0.0001a **
95% CI	67.3 to 72.8	68.3 to 70.9	76.6 to 79.7	
		vs control, *p* = 0.7020c	vs control, *p* < 0.0001c ##	
			vs PG, *p* < 0.0001c ##	
Sex				
Male, n (%)	39 (29)	156 (49)	59 (47)	0.0004b **
Female, n (%)	94 (71)	160 (51)	68 (53)	
		vs control, *p* = 0.0001d ##	vs control, *p* = 0.0049d #	
			vs PG, *p* = 0.6004d	
BCVA (logMAR)				
Mean ± SD	0.04 ± 0.11	0.18 ± 0.38	0.25 ± 0.37	<0.0001a **
95% CI	0.06 to 0.01	0.22 to 0.14	0.31 to 0.18	
		vs control, *p* < 0.0001c ##	vs control, *p* < 0.0001c ##	
			vs PG, *p* = 0.0517c	
IOP (mmHg)				
Mean ± SD	14.4 ± 2.7	13.8 ± 4.3	13.9 ± 5.9	0.4169a
95% CI	13.9 to 14.9	13.3 to 14.2	12.9 to 15.0	
		vs control, *p* = 0.1872c	vs control, *p* = 0.3872c	
			vs PG, *p* = 0.7730c	
Highest IOP (mmHg)				
Mean ± SD	15.2 ± 2.6	21.0 ± 7.9	27.9 ± 12.9	<0.0001a **
95% CI	14.8 to 15.7	20.2 to 21.9	25.6 to 30.1	
		vs control, *p* < 0.0001c ##	vs control, *p* < 0.0001c ##	
			vs PG, *p* < 0.0001c ##	
No. glaucoma medications				
Mean ± SD	0	1.9 ± 1.4	1.7 ± 1.4	<0.0001a **
95% CI		1.8 to 2.1	1.5 to 1.9	
		vs control, *p* < 0.0001c ##	vs control, *p* < 0.0001c ##	
			vs PG, *p* = 0.0428c	
MD (dB)	-			
		−13.4 ± 9.2	−15.4 ± 10.4	0.0216a *
		−14.1 to −12.0	−17.2 to 13.5	
Pseudophakia				
Yes, n (%)	32 (24%)	163 (52%)	98 (77%)	<0.0001b **
No, n (%)	101 (76)	153 (48)	29 (23)	
		vs control, *p* < 0.0001d ##	vs control, *p* < 0.0001d ##	
			vs PG, *p* < 0.0001d ##	
Current smoking				
Yes, n (%)	14 (11)	34 (11)	10 (8)	0.6464b
No, n (%)	119 (89)	282 (89)	117 (92)	
		vs control, *p* = 1.0000d	vs control, *p* = 0.5241d	
			vs PG, *p* = 0.4822d	
Diabetes				
Yes, n (%)	32 (24%)	40 (13%)	16 (13%)	0.0058b **
No, n (%)	101 (76)	276 (87)	111 (87)	
		vs control, *p* = 0.0045d #	vs control, *p* = 0.0245d	
			vs PG, *p* = 1.000d	
Hypertension				
Yes, n (%)	52 (39%)	164 (52%)	73 (58%)	0.0079b **
No, n (%)	81 (61)	152 (48)	54 (42)	
		vs control, *p* = 0.0172d	vs control, *p* = 0.0042d ##	
			vs PG, *p* = 0.2944d	

*p* values are calculated by one-way analysis of variance (ANOVA) (a) or the G-test (b). Post-hoc pair comparisons were performed using the *t*-test (c) or Fisher’s exact probability test (d). The ** corresponds to the significance level at 1% (*p* < 0.01) for ANOVA or the G-test. The # and ## correspond to the significance levels at 5% (*p* < 0.0167) and 1% (*p* < 0.0033), respectively, by Bonferroni correction for multiple comparisons by the unpaired *t*-test or Fisher’s exact probability test among the three groups. PG, primary open-angle glaucoma group; EG, exfoliation glaucoma group; SD, standard deviation; CI, confidence interval; BCVA, best-corrected visual acuity; logMAR, logarithm of minimum angle of resolution; IOP, intraocular pressure; MD, visual field mean deviation; dB, decibel.

**Table 2:** Comparisons of advanced glycation end products among three groups.

Group	Control	PG	EG	*p* Value
Mean ± SD	0.56 ± 0.15	0.56 ± 0.11	0.61 ± 0.11	<0.0001a **
95% CI	0.54 to 0.59	0.54 to 0.57	0.60 to 0.63	
		vs control, *p* = 0.5120b	vs control, *p* = 0.0007b ##	
			vs PG, *p* < 0.0001b ##	

*p* values are calculated by one-way analysis of variance (ANOVA) (a). Post-hoc pair comparisons are performed using the unpaired *t*-test (b). The ** corresponds to the significance level at 1% (*p* < 0.01) for ANOVA. The ## correspond to the significance levels at 1% (*p* < 0.0033) by Bonferroni correction for multiple comparisons by the unpaired t-test among the three groups. SD, standard deviation; CI, confidence interval; PG, primary open-angle glaucoma group; EG, exfoliation glaucoma group.

**Table 3:** Possible associations among advanced glycation end products and various continuous parameters.

Parameters	*r*	Lower 95% CI	Upper 95% CI	*p* Value
Age (years)	0.11	0.03	0.19	0.0063 *
BCVA (logMAR)	0.04	−0.43	0.12	0.3540
IOP (mmHg)	−0.02	−0.10	0.06	0.6473
Highest IOP (mmHg)	0.00	−0.06	0.11	0.5616
No. glaucoma medications	0.03	−0.05	0.11	0.4942

The correlation coefficient (*r*) is Pearson’s correlation coefficient. * *p* < 0.05. CI, confidence interval; BCVA, best-corrected visual acuity; logMAR, logarithm of minimum angle of resolution; IOP, intraocular pressure.

**Table 4:** Possible association among advanced glycation end products and various categorical parameters.

Parameters	Mean ± SD (95% CI)	Mean ± SD (95% CI)	*p* Value
Sex	Male, 0.59 ± 0.13 (0.58 to 0.61)	Female, 0.55 ± 0.11 (0.54 to 0.57)	<0.0001 **
Pseudophakia	No, 0.56 ± 0.12 (0.55 to 0.58)	Yes, 0.58 ± 0.12 (0.57 to 0.59)	0.0384 *
Pseudoexfoliation	No, 0.56 ± 0.12 (0.55 to 0.57)	Yes, 0.61 ± 0.11 (0.59 to 0.63)	<0.0001 **
Current smoking status	No, 0.58 ± 0.12 (0.57 to 0.59)	Yes, 0.52 ± 0.11 (0.50 to 0.55)	0.0012 **
Diabetes	No, 0.57 ± 0.16 (0.59 to 0.58)	Yes, 0.59 ± 0.15 (0.56 to 0.63)	0.0377 *
Hypertension	No, 0.56 ± 0.13 (0.54 to 0.57)	Yes, 0.58 ± 0.11 (0.57 to 0.59)	0.0151*

The *p* values are calculated using the *t*-test between groups. * *p* < 0.05, ** *p* < 0.01. SD, standard deviation; CI, confidence interval.

**Table 5:** Possible associations among advanced glycation end products and various parameters analyzed by a multiple regression model.

Parameters	*r*	Lower 95% CI	Upper 95% CI	*p* Value	Standard β
Age (years)	0.00	0.00	0.00	0.6048	0.03
Sex (male)	0.03	0.02	0.04	<0.0001 **	0.23
BCVA (logMAR)	0.00	-0.03	0.03	0.8099	0.01
IOP (mmHg)	0.00	0.00	0.00	0.6698	−0.02
Highest IOP (mmHg)	0.00	0.00	0.00	0.1129	−0.08
No. glaucoma medications	0.00	0.00	0.01	0.3504	0.05
Pseudophakia (yes)	0.00	−0.01	0.01	0.7486	0.02
PG (/control)	−0.03	−0.04	−0.01	0.0002 **	−0.18
EG (/control)	0.03	0.02	0.05	0.0004 **	0.19
Current smoking (yes)	−0.04	−0.06	−0.02	<0.0001 **	−0.19
Diabetes (yes)	0.02	0.00	0.03	0.0311 *	0.09
Hypertension (yes)	0.01	−0.01	0.02	0.3190	0.04

*p* values are calculated using a multiple regression model. * *p* < 0.05 and ** *p* < 0.01. R, regression coefficient; CI, confidence interval; BCVA, best-corrected visual acuity; logMAR, logarithm of minimum angle of resolution; IOP, intraocular pressure; PG, primary open-angle glaucoma group; EG, exfoliation glaucoma group.

## Supplementary Materials

The following are available online at https://www.mdpi.com/2076-3921/9/8/755/s1, Figure S1: Distribution of advanced glycation end products (AGEs) in each disease group.
