# Supplementary material for: Advanced Glycation End Product Accumulation in Subjects with Open-Angle Glaucoma with and without Exfoliation
Source: Antioxidants (Basel). 2020 Aug 15;9(8):755. doi: 10.3390/antiox9080755 (PMC7465686; doi:10.3390/antiox9080755)
Supplement: Supplementary file 1 [file antioxidants-09-00755-s001.pdf]

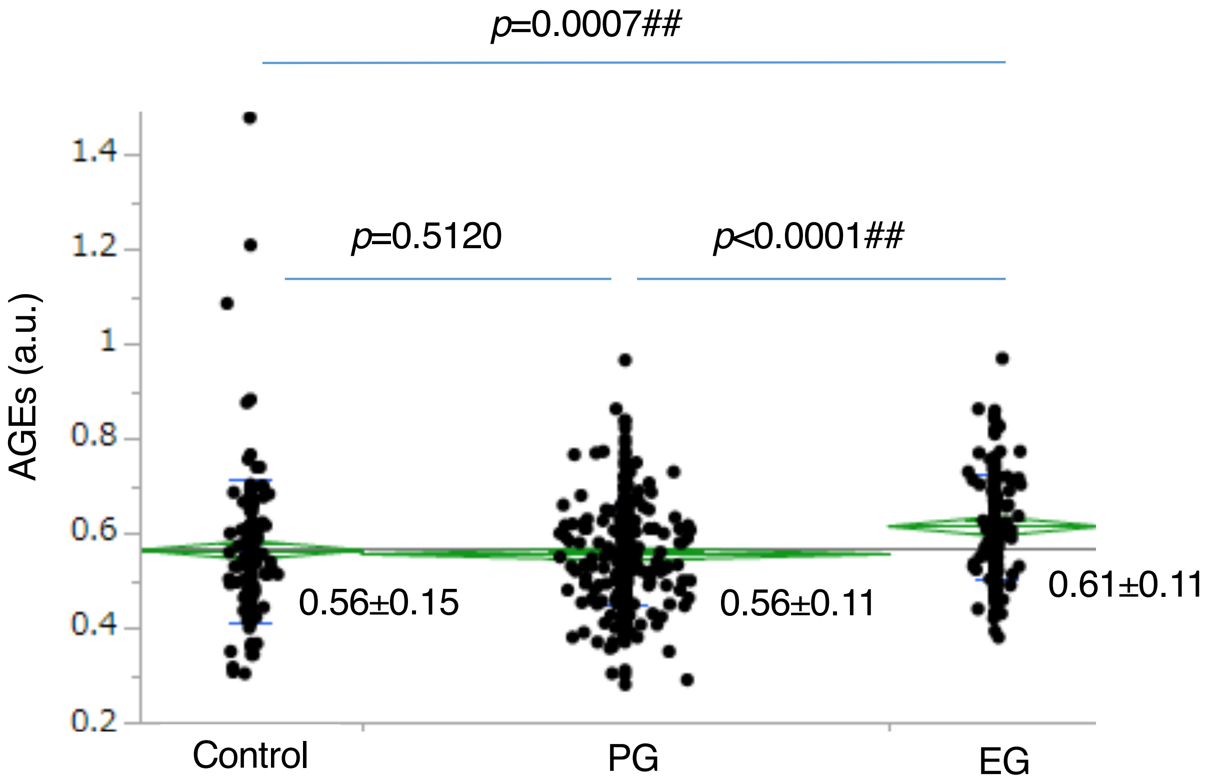

Figure S1. Distribution of advanced glycation endproducts (AGEs) in each disease group. The *p* values of the post-hoc pair comparisons are calculated by the unpaired t-test. The ## correspond to the significance levels at 1% ($p<0.0033$) by Bonferroni correction for multiple comparisons by the unpaired t-test among three comparison groups. PG, primary open-angle glaucoma group; EG, exfoliation glaucoma group; a.u., arbitrary units.
